# Anti-Calcitonin Gene-Related Peptide Monoclonal Antibody Is Effective for Preventing Migraine Aura Without Headache

**DOI:** 10.3390/neurolint16060097

**Published:** 2024-10-29

**Authors:** Yasushi Shibata

**Affiliations:** Department of Neurosurgery, Headache Clinic, Mito Medical Center, University of Tsukuba, Ibaraki 310-0015, Japan; yshibata@md.tsukuba.ac.jp; Tel.: +81-29231-2371

**Keywords:** aura, migraine, cortical spreading depression, calcitonin gene-related peptide

## Abstract

Background: Anti-calcitonin gene-related peptide monoclonal antibodies (CGRP mAbs) are clinically effective in preventing the migraine attacks, photophobia, and migraine auras associated with headaches. However, no study has yet investigated the effectiveness of CGRP mAbs in preventing migraine aura without headache. Case report: A female patient of 49 years old presented with a long history (since age 10) of photosensitivity and typical migraine auras without a headache. The symptoms slightly responded to oral medication, lomerizine chloride, but did not completely resolve. Just one day after the administration of galcanezumab, her photo-hypersensitivity and migraine aura had completely resolved. Consequently, the administration of the oral migraine preventive medication was discontinued. Monthly galcanezumab at a dose of 120 mg was continuously given and she did not re-experience any auras or headaches. Conclusions: The use of CGRP mAbs can be considered as a potential treatment in preventing migraine aura without headache. Currently, CGRP mAb is indicated only for migraines with and without auras. Given our findings and the promising effects of this medication for this migraine subtype, a large clinical trial is required to better assess the effects and potential adverse events of CGRP mAb in patients with migraine aura without headache.

## 1. Introduction

Migraine is a highly prevalent and disabling headache disorder. Due to the severity of the headache, patients with migraine may struggle to lead a normal life. Migraine is commonly seen in working-age individuals, particularly in their 30 s and 40 s, which contributes to a significant economic burden. The patients with migraines frequently experience hypersensitivity to light, sound and odors. Nausea and vomiting are also common symptoms in migraine sufferers. Some migraine patients experience an aura just before the onset of a migraine headache. Aura symptoms may include visual, sensory, speech and motor disturbances. Typical visual auras are scintillating scotoma and visual fields distortions. Cortical spreading depression (CSD) has been demonstrated as the pathology of migraine aura [1].

Migraine aura without headache is classified as the subtype of migraine with aura in the International Classification of Headache Disorders, 3rd edition [2]. Several case reports of the patients of aura without headache have been reported [3,4]. Even though these patients did not experience any headaches, their aura symptoms significantly disturbed their daily lives. Clinical studies on migraine auras without headaches are limited, and the standard management for this condition has not been extensively researched.

The prevalence of auras without headaches has been reported as 7 (0.175%) in 4000 in the general population of Denmark [5]. Due to its rarity, other prevalence studies of auras without headaches in the general population have not been reported.

Patients with auras without headaches do not think of this symptom as a migraine; thus, they may initially seek treatment from ophthalmology clinics. A study from a United State ophthalmology clinic reported that 65 out of 1000 patients (6.5%) have experienced migraine auras without headaches [6]. In Japanese ophthalmology clinics, 35 out of 1063 individuals (3.2%) were diagnosed as having auras without headaches [7]. In this study, the age of the patients with auras without headaches showed a biphasic distribution, dominant in the second to third and sixth decades. The Japanese nationwide survey demonstrated that the prevalence of migraine is dominant in the third and fourth decades [8]. Therefore, the age distribution of auras without headaches has an additional peak in older populations.

Other studies showed that migraine auras without headaches tend to occur later in life and are more common in elderly patients [9]. Late-life migraine accompaniments have been reported in ages ranging from 40 to 73 years [10]. These symptoms included visual, speech, sensory, and motor disturbances. Headaches occurred in association with the aura episodes in only 40% of cases. Most of these auras were transient visual symptoms; therefore, these should be differentiated from transient ischemic attacks or epilepsy. Case reports of patients with auras without headaches who have been misdiagnosed as having had transient ischemic attacks have been frequently reported [4].

Calcitonin gene-related peptide (CGRP) has been studied as a key neurotransmitter involved in migraines. Targeting CGRP by blocking its action has been explored as a potential migraine therapy. Anti-calcitonin gene-related peptide monoclonal antibody (CGRP mAb) and CGRP receptor antagonist (gepant) are clinically effective in preventing migraine attacks, photophobia, and migraine auras associated with headaches. In one study, 44% (69/158) of migraine patients showed more than a 50% response rate after six months of treatment with CGRP mAbs. Significant decreases in photophobia (−19.5%, *p* < 0.001) and aura ratios (−25.1%, *p* = 0.008) were also found in a greater than 50% response rate group [11].

Some basic studies have revealed that CGRP mAb and gepant are effective in the inhibition of CSD [1]. However, no clinical study has yet investigated the effectiveness of CGRP mAb in the prevention of migraine auras without headaches.

## 2. Case Report

This study included female patient with a long history (since age 10) of photosensitivity and migraine auras without headaches. While she experienced typical scintillating scotomas several times per year that resolved within 2 h, mild nausea and increased photosensitivity after these migraine auras were also reported. She had also experienced mild photosensitivity almost every day without scintillating scotomas. Based on her medical history, she has been receiving anti-anxiety medication for a known panic disorder.

**Figure 1:**
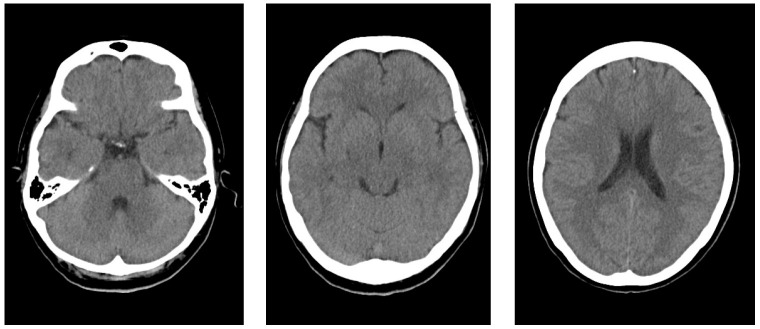
The aura symptoms showed a slight response to this therapy but did not completely resolve.

At her first medical visit to our clinic at age 49, a neurological examination and brain computed tomography showed no abnormalities (Figure 1). Routine blood tests did not indicate any abnormalities such as anemia, infection, liver, or kidney dysfunctions.

After a detailed discussion explaining the indications of CGRP mAb therapy, the patient’s approval was obtained, and 240 mg of galcanezumab was subcutaneously injected. Just one day after the administration of galcanezumab, her mild photosensitivity disappeared and the typical scintillating scotoma had completely resolved. No adverse event from the galcanezumab was observed. Consequently, the administration of the oral migraine preventive medication was discontinued. Monthly galcanezumab at the dose of 120 mg has been continuously given, as she currently wishes to continue it, and she has not experienced any auras or headaches to date. We will consider discontinuing the treatment if any issues arise.

## 3. Discussion

This case report demonstrated that CGRP mAb was effective in preventing migraine aura symptoms for the patient with migraine auras without headaches. This is the first case report to investigate the effectiveness of CGRP mAb for migraine auras without headaches. Typically, oral migraine preventive medications are effective for migraine auras without headaches [4,9]; however, some patients tend to exhibit resistance to these therapies. In such cases, the use of CGRP mAb is considered an alternative treatment option, with reasonable evidence supporting its efficacy not only in reducing migraine attacks but also in alleviating associated migraine auras and photohypersensitivities [11,12]. There is also a study indicating that the use of CGRP mAb may improve sleep in patients with migraines [13].

In Japan, calcium-channel blockers are the most commonly used first-line preventive medication for migraine [14]; lomerizine chloride is one of the most commonly used oral preventive calcium-channel blockers in Japan. This medication is commercially available only in Japan. The main advantage of lomerizine chloride is its minimal adverse effects compared to other drugs like valproic acid, amitriptyline, or propranolol. Lomerizine chloride has little to no effect on systemic blood pressure. Additionally, in Japan, topiramate and botulinum toxin are not covered by health insurance for the preventive treatment of migraines.

There is abundant evidence that CSD is responsible for neuronal inflammation and the activation of the central and peripheral trigeminal nerves in migraines. During CSD, neurons, glia, and vascular cells locally release adenosine triphosphate (ATP), glutamate, potassium, and hydrogen ions, while activated perivascular nerves release CGRP and nitrous oxide. CSD also opens neuronal pannexin1 (Panx1) mega channels, triggering the release of caspase-1 and high-mobility group box 1 (HMGB1) from neurons and activating nuclear factor kappa B (NFκB) in astrocytes [15]. These pro-inflammatory molecules diffuse toward the cortical surface, activating pial nociceptors, which lead to neurogenic inflammation, persistent activation of dural nociceptors, and the subsequent stimulation of central trigeminal neurons in the trigeminal spinal nucleus [16]. The release of these inflammatory neurotransmitters, including CGRP, into the subarachnoid space has been discussed as the cause of migraine headache. A magnetic resonance imaging study of 12 patients with auras without headaches and 45 patients with migraines with auras and headaches showed no volumetric and morphological difference in the brain and subarachnoid space [17]. Therefore, these anatomical differences are not factors that determine migraine auras and headaches. Additionally, CSD events significantly increase CGRP messenger ribonucleic acid (mRNA) levels in the rat cerebral cortex [18]. This is the one of the mechanisms that initiate and maintain elevated CGRP levels in migraine and other conditions such as post-traumatic headache.

Most migraine attacks do not involve aura symptoms. A study on cerebral blood flow found no blood flow changes during migraine attacks without auras, but did detect hypovolemia during migraines with auras [19]. In 2001, Hadjikhani and colleagues used functional magnetic resonance imaging (fMRI) to study three migraine patients while they were experiencing visual auras [20]. They observed that the blood oxygenation level-dependent (BOLD) signal in these patients progressed slowly and contiguously over the occipital cortex corresponding with the visual aura symptoms. Since the BOLD signal reflects the balance between oxygen delivery and oxygen consumption rather than blood flow itself, the researchers concluded that migraine auras are triggered not by ischemia, but by the abnormal neuronal activity [20].

Although the primary cause of CSD is not ischemia, it is reasonable to think that ischemia is the contributing factor of CSD. Migraine auras without headaches have been frequently observed in elderly patients. Elderly patients tend to have arteriosclerosis and cerebral ischemia. However, most migraine auras are completely reversible and ischemic stroke associated with migraine auras has not been reported. Kawahara et al. reported upregulated brain-derived neurotrophic factor (BDNF) mRNA levels in rat cerebral cortexes following CSD [21]. The increased BDNF following CSD in the cortex was consistent with the involvement of BDNF in cortical ischemic tolerance. Thus, the ischemia may be one condition contributing to the initiation of CSD, and CSD itself may protect the cerebral cortex from ischemic damage. CGRP and nitrous oxide are released following CSD; these neurotransmitters dilate cerebral blood vessels, providing a protective effect against cerebral ischemia.

A basic study has reported on the inhibitory effects of CGRP mAb and gepant on CSD [1]. Cortical slice studies showed that the blockage of CGRP receptors in vitro resulted in the inhibition of CSD [22]. CGRP mAbs do not cross the blood–brain barrier. Therefore, they are thought to act on the trigeminal nerve outside of this barrier. Our case suggests that stabilizing the trigeminal nerve with CGRP mAb is sufficient to prevent auras and CSD in patients with aura without headaches, at least at the clinical level.

Whitty reported 16 case reports of migraine auras without headaches. In nine cases, the patients had attacks of both auras and headaches and auras alone, and seven attacks were accompanied by auras but no headaches [3]. Six of the nine cases in the first group show a sequence of early typical migraine followed, later in life, by auras alone, sometimes with an intermediate period in which the headaches became gradually less prominent. CGRP concentration decreases with age [9]. This may be one reason why migraine auras without headaches have been reported in relatively older patients. For these patients, migraine headaches may diminish with aging, but auras can still persist.

The prevalence of auras without headaches has been reported as 7 (0.175%) in 4000 in the general population in Denmark [5]. In this study, the participants were interviewed by a physician, because the diagnosis of migraine auras is not easy, and photophobia can easily be mistaken as auras. Out of 4000 people, 62 had attacks of migraine auras with headaches as well as migraine auras without headaches [5]. This means that CSD usually triggers migraine, but it is not always sufficient to trigger for headache.

The prevalence of auras without headaches at ophthalmology clinics in US and Japan have been reported as from 3.2 to 6.5% [6,7]. These studies used self-reported written questionnaires as surveys. Therefore, this diagnosis may not be accurate. Patients with auras without headaches have been reported more frequently in ophthalmology clinics than in neurology or general medicine clinics. There may be many potential patients who are not adequately diagnosed or managed. In the future, clinical and epidemiological studies on auras without headaches are anticipated.

The diagnosis of a migraine aura without a headache is not easy because there are no effective diagnostic examinations. The correct diagnosis heavily depends on detailed interviews of symptoms, past history, and family history. If patients have a history of migraines and/or hypersensitivity to light, sound or smell, the diagnosis of an aura without a headache may not be difficult. Differential diagnoses include transient ischemic attacks and epilepsy. The onset of transient ischemic attacks is usually sudden. There are many types of epilepsy, including sudden onset and gradual onset epilepsy. Migraine auras usually occur with a gradual onset. The symptom duration of a transient ischemic attack is relatively short, lasting 5–10 min, while migraine aura symptoms can last up to 60 min [9]. Visual auras should be differentiated from occipital lobe epilepsy. Generally, the symptoms of a visual aura last longer than occipital lobe epilepsy. Visual auras tend to be black and white, while occipital lobe epilepsies have visual auras with colors. However, these symptom differences do not definitively differentiate these conditions [9]. Neuroimaging studies and electroencephalogram studies may help to differentiate these pathologies, although certain populations of these patients show no abnormalities.

Migraines and epilepsy have tight connections in terms of clinical symptoms and pathophysiological mechanisms [23,24,25,26,27]. The incidence of epilepsy in patients with migraines is significantly higher than that in patients with tension-type headache [24]. Some patients develop epileptic seizures after migraine attacks or auras; these phenomena are known as migraine-triggered seizures or migralepsy [28,29,30,31,32].

Currently, CGRP mAb is indicated only for migraine with and without auras. Given our findings, and the promising effects of this medication for this migraine subtype, a large clinical trial is required to better assess the effects and potential adverse events of CGRP mAb in patients with migraine auras without headaches. The optimal dose and frequency of CGRP mAb injections should be investigated in the near future.

## 4. Conclusions

This study reported on a case of a patient with a visual aura without a headache. Oral migraine preventive medications were ineffective in controlling her aura symptoms, but CGRP mAb completely eliminated them.

The use of CGRP mAbs can be considered as a potential treatment method in preventing migraine auras without headaches. Currently, CGRP mAb is indicated only for migraines with and without auras. Given our findings, and the promising effects of this medication for this migraine subtype, a large clinical trial is required to better assess the effects and potential adverse events of CGRP mAb in patients with migraine auras without headaches. Although CGRP mAbs do not penetrate the blood–brain barrier, trigeminal nerve stabilization with CGRP mAb can prevent CSD.

## Data Availability

The original contributions presented in the study are included in the article, and further inquiries can be directed to the corresponding author.
